# DNA methylation as a diagnostic tool

**DOI:** 10.1186/s40478-022-01371-2

**Published:** 2022-05-08

**Authors:** Kristyn Galbraith, Matija Snuderl

**Affiliations:** 1 Department of Pathology, New York University School of Medicine, New York, NY 10016 USA; 2 Laura and Isaac Perlmutter Cancer Center, New York University Langone Medical Center, 240 E 38th Street, 22nd Floor, New York, NY 10016 USA

## Abstract

DNA methylation of cytosines in CpG sites throughout the genome is an epigenetic mark contributing to gene expression regulation. DNA methylation patterns are specific to tissue type, conserved throughout life and reflect changes during tumorigenesis. DNA methylation recently emerged as a diagnostic tool to classify tumors based on a combination of preserved developmental and mutation induced signatures. In addition to the tumor classification, DNA methylation data can also be used to evaluate copy number variation, assess promoter methylation status of specific genes, such as MGMT or MLH1, and deconvolute the tumor microenvironment, assessing the tumor immune infiltrate as a potential biomarker for immunotherapy. Here we review the role for DNA methylation in tumor diagnosis.

## Introduction

DNA methylation of cytosines in CpG sites throughout the genome is an ancient evolutionary epigenetic modification contributing to chromatin structure, gene silencing, and genetic stability. Methylation occurs at the C5 position of cytosine within CpG dinucleotides by DNA methyltransferases (DNMT3A and DNMT3B) during embryonic development. This methyl mark is maintained throughout cell divisions by a maintenance DNA methyltransferase (DNMT1), establishing an epigenetic marking of the genome. DNA methylation plays a critical role in the development of tissue-specific gene expression patterns [1]. The genome-wide DNA methylation pattern is a composite of methylation patterns of the cell of origin, as well as acquired methylation changes due to aging [17], environment [2], or mutations [26]. It has been shown that the methylation patterns of tumors remain preserved, and accurately reflect the cell of origin, remaining stable throughout the course of the disease, and rendering this a dependable biomarker for tumor classification. DNA methylation has been successfully used to further subcategorize major classes of tumors that cannot be distinguished by histology alone, for example medulloblastomas, ependymomas, and supratentorial PNETs.

Here we describe the basis and utility of the DNA methylation-based tumor classification in clinical practice and how it has been implemented in the most recent WHO Classification of Central Nervous System Tumors, 5^th^ edition. We also describe other applications such as copy number and MGMT promoter analysis for brain tumor molecular testing. In addition, we discuss deconvolution of bulk DNA methylation data in the evaluation of the tumor microenvironment.

### Technical aspects of DNA methylation

Genome-wide DNA methylation analysis can be performed using a variety of analytical platforms, either sequencing or array based. Whole genome bisulfite sequencing, targeted bisulfite sequencing such as TruSeq Methyl Capture, and DNA methylation array represent the three most common approaches. Less commonly used is DNA methylation assessment using long reads such as Nanopore sequencing [11, 12]. While the whole genome bisulfite sequencing is the most comprehensive, it requires high-quality DNA, it is costly, and it is not suitable for formalin-fixed paraffin-embedded (FFPE) material. Similarly, targeted methylation sequencing, such as TrueSeq MethylCapture, while evaluating over 3.3 CpGs is not FFPE compatible. The DNA methylation EPIC array (Illumina, Inc, CA) has emerged as a dominant molecular assay for genome-wide analysis of DNA methylation in FFPE tissue. In addition to FFPE compatibility, array requires a relatively low starting DNA input (250 ng of DNA input compared to 500 ng for targeted MethylCapture sequencing) and carries a lower cost. DNA methylation array analysis is a well-established four-day process [24]. DNA can be extracted using any clinical method of DNA isolation. The DNA is quantified using a Qubit 2.0 fluorometer from Life Technologies along with the Qubit dsDNA BR Assay kit. On day one, bisulfite conversion is performed using the EX-96 DNA Methylation kit from Zymo research. If samples consist of formalin-fixed paraffin-embedded tissue, then the degraded FFPE DNA should be restored using the Infinium HD FFPE Restore kit from Illumina. Array hybridized DNA is scanned and raw data files with the fluorescence intensity data for each probe are produced by the iScan system for analysis. The data is then processed through customized bioinformatics pipelines including removal of poorly performing, SNP, and sex chromosome probes, and, if required, batch corrections and normalization for differential methylation and other analyses [6].

### The rise of DNA methylation classifiers

**Fig. 1:**
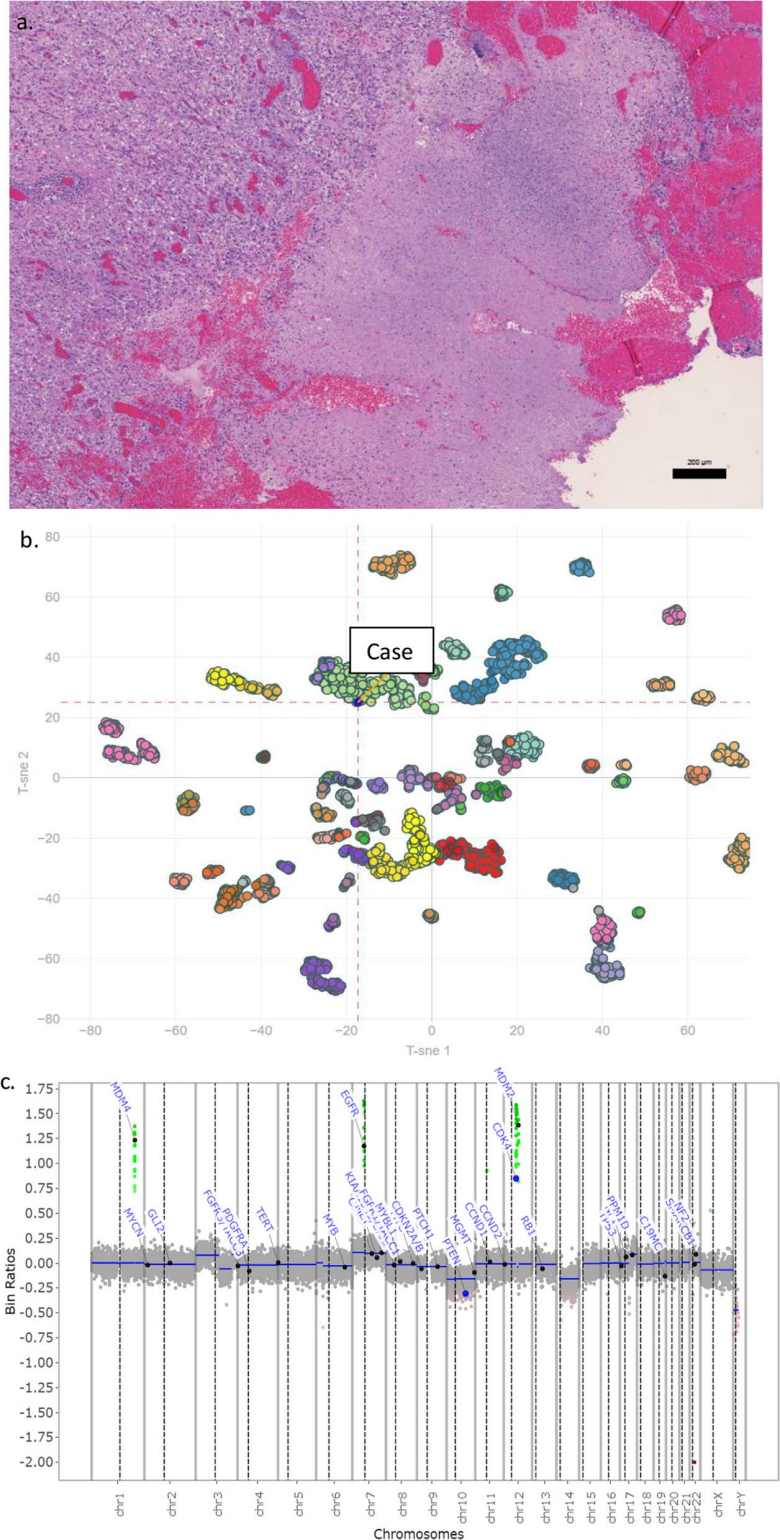
Diagnostically and clinically useful DNA methylation report with an indeterminate score. A hematoxylin and eosin stained section of a glioblastoma with about 50% cellularity (**a**). Classified by DNA methylation as a glioblastoma subclass mesenchymal with an indeterminate score of 0.569. T-SNE shows this case clustering closely with glioblastoma, subclass mesenchymal in green (**b**). The copy number plot generated from the DNA methylation data shows an EGFR amplification, consistent with the diagnosis of glioblastoma, as well as MDM4, MDM2, and CDK4 amplifications (**c**)

While the DNA methylation array has been embedded in TCGA and other consortia analyses since the first iteration of DNA methylation arrays, it has been only recently that DNA methylation signatures were used for generation of tumor-specific signatures, probability scores, and diagnoses. Capper et al. have shown that brain tumors can be accurately subclassified using DNA methylation signatures and a machine learning algorithm such as the Random Forest classifier [6]. The result of the Classifier is represented as a calibrated score, representing a probability that the tumor belongs in a given subclass. It has been established that a threshold score of greater than 0.9 has to be reached in order to achieve sensitivity of 0.989 and specificity of 0.999 [6]. Some tumors are further stratified into subclasses for which a calibrated score has to be higher than 0.5. For a methylation class, a calibrated classifier score between 0.3 and 0.9 is considered indeterminate. Nevertheless, the calibrated score less than 0.9 may still be informative, particularly in tumors with a low tumor cell content, where tumor DNA methylation signature may be diluted by normal brain or inflammatory cells. These cases require close collaboration between neuropathology and molecular pathology, sometimes with additional molecular techniques to resolve controversial diagnoses. For example, a case with a low tumor cell content may still provide diagnostic or clinical utility in copy number aberrations provided by the DNA methylation data (Fig. 1). A case with a high tumor cell content but a score between 0.5 and 0.8 should not be reported but may require further molecular evaluation, including RNA or DNA sequencing since low calibrated scores in cases with high tumor cell content often suggest a rare or novel driver, as seen in NTRK-driven gliomas [29]. A calibrated score below 0.3 is considered negative, suggesting that DNA methylation is not a useful diagnostic tool and results should not be reported.

### DNA methylation classifier as a diagnostic tool

Since the TCGA analyses of glioblastoma and the molecular classification of medulloblastoma, it has been recognized that tumors with similar histopathology can be divided into molecularly and clinically distinct subgroups [31, 28]. Prior to DNA methylation, CNS primitive neuroectodermal tumors (CNS-PNETs) was a broad category of tumors characterized by small, poorly differentiated, embryonal appearing cells with both glial and neuronal differentiation. DNA methylation profiling of 323 tumors that were histologically deemed PNETs revealed that all but 77 tumors classified as different entities, for example, medulloblastoma, high grade glioma, ependymomas, and pineal tumors [25]. But these 77 tumors that did not classify with known tumors formed 4 distinct clusters, now known as CNS neuroblastoma with *FOXR2* activation, CNS Ewing sarcoma family tumor with *CIC* alteration, CNS high-grade neuroepithelial tumor with *MN1* alteration, and CNS high-grade neuroepithelial tumor with *BCOR* alteration, all driven by specific gene fusions [25]. DNA methylation has also shown epigenetically different subgroups in genetically less heterogenous tumors, such as atypical teratoid rhabdoid tumors (ATRTs) that are characterized by the loss of SMARCB1 [19]. Three distinct subgroups of ATRTs, almost all with loss of SMARCB1, were discovered using DNA methylation analysis, each with differing locations and distinct and possibly targetable pathways: ATRT-TYR overexpressing tyrosinase, ATRT-SHH with signaling involving the SHH pathway, and ATRT-MYC with overexpression of the MYC oncogene [19]. DNA methylation has also been used to more accurately classify ependymomas and subependymomas into the following 8 categories: supratentorial subependymoma, supratentorial ependymoma, posterior fossa subependymoma, posterior fossa ependymoma type A, posterior fossa ependymoma type B, spinal subependymoma, spinal myxopapillary ependymoma, and spinal ependymoma. In addition, Witt et al. found that many histologically diagnosed ependymomas were more accurately classified by DNA methylation as subependymomas or spinal myxopapillary ependymomas [32]. Accurately stratifying subependymomas, ependymomas, and myxopapillary ependymomas is crucial due to differences in treatment and prognosis. DNA methylation is useful in accurately subclassifying tumors with indistinct morphologies.

DNA methylation may also be useful as a prognostic tool in meningiomas, more precisely than by the current CNS WHO grading classification based on histology alone. DNA methylation of meningiomas found two major epigenetic groups with distinct prognostic implications [23]. Grade 1 meningiomas were in what they deemed group A and grade 3 meningiomas were primarily in what they deemed group B, while grade 2 meningiomas were scattered between both groups. Looking at progression free survival, histologically defined grade 1 meningiomas that classified in a higher-grade group by DNA methylation behaved similarly to grade 2 meningiomas; conversely, histologically defined grade 2 meningiomas that classified in a lower grade group by DNA methylation behaved similarly to grade 1 meningiomas [23]. Research has shown that while DNA methylation is useful in prognostication of meningiomas; it alone may not be sufficient. Nassiri et al. found that unsupervised clustering of copy number variation, whole exome sequencing, DNA methylation, and RNA sequencing data in isolation resulted in 6 stable subgroups from each data type; however, the clusters across data types were not identical or significant and outcome associations were unique for each data type [21]. Combining this data and running cluster on cluster analysis revealed four stable subgroups that correlated well with recurrence free survival, suggesting that the combination of molecular data provides the most accurate prognostic information [21].

DNA methylation has great utility and clinical application, arguably the most important being it’s overall impact on diagnosis. Studies have shown that using DNA methylation as a diagnostic tool results in more accurate classification of tumors than by histology alone, altering the diagnosis in 12% of cases as described by one study [6]. Additional research shows that in challenging cases where the diagnosis is often descriptive, DNA methylation can provide an answer 50% of the time [33]. Most notably, one study, focused on diagnostically challenging cases, showed that the addition of DNA methylation resulted in the direct change in patient management in 15% of cases [20].

Clinical trials, and pediatric brain tumors in particular, due to the diagnostic difficulties and low number of cases, are especially sensitive to the enrollment of misdiagnosed patients. DNA methylation-based re-analysis of the Children’s Oncology Group ACNS0332 CNS-PNET Trial showed that 71% of histologically confirmed PNETs actually represented other molecularly defined brain tumor entities that should have been excluded from trials, ultimately leading to trial failure [18]. Therefore, it is paramount that brain tumor clinical trials incorporate DNA methylation as a molecular screening assay to assure the accuracy of diagnostics. Since EPIC array is highly robust with a uniform data format as well as reproducible between the laboratories [6], screening for clinical trials does not require a centralized laboratory and can be performed at any clinical laboratory with validated DNA methylation array testing.

### Copy number analysis using DNA methylation

In addition to the classifier, copy number data can also be generated from the DNA methylation array data. The raw signal intensity data from the DNA methylation array can be analyzed through the conumee package using R [6]. In DNA methylation analysis, every CpG analyzed is represented by either a probe for methylated or a probe for unmethylated. In copy number analysis, the signal intensities of the methylated and unmethylated probes are summated and compared against healthy reference samples with no copy number variations and then plotted by chromosomal location. A high copy number ratio correlates with an amplification or a trisomy, a low copy number ratio correlates with a deletion [6]. Putative gene fusions can also be found if they are associated with DNA breaks and microdeletions [22]. One of the first papers subclassifying glioblastomas by DNA methylation, also used copy number data derived from DNA methylation data to further characterize these subgroups [26]. Sturm et al. have shown that the cluster of RTKI glioblastomas commonly had PDGFRA amplifications and the cluster of RTKII glioblastomas carried whole chromosome 7 gain and whole chromosome 10 loss, CDKN2A homozygous deletion, and EGFR amplification [26]. Haider et al. used DNA methylation array data to analyze copy number variation profiles in T-cell lymphoblastic leukemia and lymphoma and found 17 different chromosomal regions with recurrent copy number variations, including a gain in chromosome 5p and a deletion in chromosome 13q that were significantly more prevalent in T-lymphoblastic lymphoma as compared to T-lymphoblastic leukemia [15]. These findings were confirmed using single nucleotide polymorphism (SNP)-array analysis and results were concordant, supporting the use of DNA methylation array data in copy number variation analysis. Studies have shown reproducibility of results within analysis of copy number by DNA methylation as well as comparability to CNV analysis across different SNP array platforms. In addition, DNA methylation arrays have coverage of different gene regions than SNP arrays, allowing for the detection of alterations by DNA methylation that were not detected by SNP array [9]. This enables detection of non-coding regulatory regions as putative drivers. Vasudevaraja et al. have shown in focal cortical dysplasia samples that amplifications of EGFR enhancer regions or PDGFRA promoter regions were associated with high expression of EGFR, and PDGFRA in neurons, respectively [30].

### MGMT methylation status analysis

Glioblastoma is the most primary malignant brain tumor in adults with the current standard of care being surgical excision followed by temozolomide and radiation therapy. The addition of temozolomide has a survival benefit to some patients, but not all, a molecular biomarker to successfully predict patient response is needed. MGMT promoter hypermethylation has been shown to promote sensitivity to temozolomide and can be used to help predict response to treatment [5]. The MGMT methylation status can be obtained directly from the array data using the MGMT-STP27 model and is highly concordant with the MGMT pyrosequencing results [3]. Similarly, DNA methylation analysis can also be used for analysis of other cancer relevant promoters, such as MLH1 [4].

### Tumor microenvironment analysis from the whole tumor DNA methylation

Tumors have been recognized to have complexity matching, if not exceeding, that of normal tissues. The biology of tumors can only be understood if the tumor cells as well as the microenvironment they create are examined. Cancer cells, endothelial cells, pericytes, and immune cells all play a role in the progression of disease [16]. T-cells perform cancer immune surveillance at early stages of premalignant lesions; however, eventually the immune system is overcome and cancer cells are able to not only avoid immune surveillance but actively suppress it [8]. Tumor cells recruit, T regulatory cells, tumor associated macrophages (TAMs), and myeloid derived suppressor cells, cells that function in interfering with the effector T-cells that kill tumor cells [13]. Immune checkpoint inhibitors to PD-1, PD-L1, and CTLA-4, show varied responses dependent on tumor type. Data from clinical trials has shown that cancers with increased lymphocyte infiltration show an improved response rate to immunotherapies [7]. Determining which patients would benefit from immunotherapies based on the tumor microenvironment is crucial to maximize efficacy and can be done using CIBERSORT-based deconvolution to genome-wide DNA methylation data from whole tumor tissue (known as MethylCIBERSORT). Many studies have used MethylCIBERSORT as a tool to evaluate the microenvironment of different tumors. Tang et al. used MethylCIBERSORT to evaluate the tumor microenvironment of pleomorphic xanthoastrocytomas (PXAs) and found that compared to gangliogliomas, PXAs have significantly increased CD8 T-cell epigenetic signatures comparatively, suggesting the potential for success with immunotherapy treatments in these tumors [27]. Grabovska et al. used MethylCIBERSORT to evaluate the tumor microenvironment of over 6,000 central nervous system tumors and found three broad immune clusters with distinct tumor subtypes, molecular subgroups, and prognosis [14]. Cui et al. have utilized MethylCIBERSORT to deconvolute tumor microenvironment across molecular subtypes of gliomas [10].

## Conclusion

DNA methylation array is a 4 day process that can be performed on formalin fixed and paraffin embedded tissue and has a multitude of uses in the diagnostic and clinical settings. Many studies have shown the utility of DNA methylation array data in more accurately classifying difficult to diagnose brain tumors as well as subclassifying histologically similar brain tumors, both important factors in treatment of the patient as well as accurate allocation of cases in the clinical trial setting. In the more accurate classification of brain tumors by DNA methylation array, new tumor entities such as polymorphous low grade tumor of the young and high grade astrocytoma with piloid features have been included in the most recent iteration of the World Health Organization classification of central nervous system tumors. In addition to the classifier, the data gleaned from the DNA methylation array can also be used to generate copy number data as well as evaluate the tumor microenvironment, both directly impacting treatment. DNA methylation can also be used for specific biomarkers and cancer-relevant promoters such as MGMT promoter methylation status and MLH1 status, respectively. DNA methylation is a robust method with a variety of diagnostic and clinical uses. 

## Data Availability

N/A.
